# Effect of Silane Coupling Agent on Tribological Properties of Hemp Fiber-Reinforced Plant-Derived Polyamide 1010 Biomass Composites

**DOI:** 10.3390/ma10091040

**Published:** 2017-09-05

**Authors:** Yosuke Nishitani, Tetsuto Kajiyama, Toshiyuki Yamanaka

**Affiliations:** 1 Department of Mechanical Engineering, Faculty of Engineering, Kogakuin University, 2665-1 Nakano, Hachioji, Tokyo 192-0015, Japan; 2 Jonan Branch, Tokyo Metropolitan Industrial Technology Research Institute, 1-20-20, Minamikamata, Ota, Tokyo 144-0035, Japan; kajiyama.tetsuto@iri-tokyo.jp; 3 Tokyo Metropolitan Industrial Technology Research Institute, 2-4-10, Aomi, Kotoku, Tokyo 135-0064, Japan; yamanaka.toshiyuki@iri-tokyo.jp

**Keywords:** polymer-matrix composites, friction, wear, plant-derived polyamide, hemp fiber, silane coupling agent

## Abstract

We have studied the effects of silane coupling agents used for the surface treatment of fiber on the tribological properties of hemp fiber (HF) reinforced plant-derived polyamide 1010 (PA1010) biomass composites. Hemp fibers were surface-treated by two surface treatment methods: (a) alkali treatment by sodium hydroxide solution and (b) surface treatment by silane coupling agents. Three types of silane coupling agents, namely aminosilane, epoxysilane and ureidosilane were used. These HF/PA1010 biomass composites were extruded using a twin extruder, and injection-molded. The mechanical and tribological properties were evaluated by the ring-on-plate type sliding wear test. It was found that tribological properties of HF/PA1010 biomass composites improved with the surface treatment by the silane coupling agent. This may be attributed to the change in the mode of friction and wear mechanism by the interfacial adhesion between fiber and matrix polymer according to the type of silane coupling agent used. In particular, the ureidosilane coupling agent showed the best improvement effect for the tribological properties of these biomass composites in this study.

## 1. Introduction

Natural fibers, such as bamboo, banana, flax, hemp, jute, kenaf, ramie, and sisal fibers, used for reinforcing polymer composites, are attracting considerable attention from industry [1,2,3,4,5,6,7]. These natural fibers have unique ecological advantages over inorganic fibers such as glass and carbon fibers because they are renewable, have relatively high strength, stiffness, low density, are low cost, biodegradable, and can be incinerated [8]. Recently these natural fiber-reinforced polymer composites have been widely used as materials in various applications [1,2,3,4]. Until now, little attention had been given to the use of such composites in the field of polymeric tribomaterials [9,10,11,12] (for mechanical sliding parts such as bearing, cum, gear, and seal). However studies in this field are gradually increasing in recent years [13,14,15,16,17,18,19,20,21,22]. These studies report that tribological properties such as the frictional and wear properties of such composites basically improve when filled with natural fibers, but study results show different tendencies according to the type of natural fiber, matrix polymer, tribo-testing mode, various test conditions, etc. However, it should be noted that most of these studies use the polymer made from petroleum as the matrix polymer of such composites.

To further enhance the eco-friendliness of materials, there is a strong need to use not only reinforcement fibers but also biopolymers obtained from plant-derived materials as the matrix polymer of these composites. Biomass polymer composites, which are used in both reinforcement fiber and matrix polymer based on renewable raw materials, are environmentally friendly to a large extent and have unique performances. Studies have been conducted on the processing, mechanical, chemical, and thermal properties of these biomass polymer composites over the last two decades [4,8,23,24,25,26,27]. In particular, the mechanical properties of natural fiber-reinforced polylactide acid (PLA) composites have been studied extensively. On the contrary, only a few studies have been published on the tribological properties of biomass polymer composites used for reinforcement fibers and matrix polymers based on renewable raw materials. Bajpai et al. reported the tribological properties of three different types of natural fiber (nettle, grewia optiva and sisal) reinforced polylactic acid (PLA) biomass composites [28]. This study concluded that the incorporation of natural fiber mats into a PLA matrix significantly improves the wear behavior of neat polymer. Therefore, to further enhance the eco-friendliness of materials in the field of tribology, it is crucial to investigate the tribological properties of various natural fiber-reinforced biopolymers, other than PLA, biomass composites.

Moreover, since the main disadvantage of such composites is the poor interfacial adhesion between natural fiber and matrix polymer, these composites exhibit poor mechanical properties [4,13,29]. Interfacial adhesion and the mechanical properties of these biomass composites can be considerably improved by suitable surface treatments. Most natural fibers are pretreated before they are used as secondary phases in composite materials. Physical and chemical methods for the surface modification of natural fibers can be used to optimize the interface between fiber and polymer. For example, physical methods such as the corona treatment and plasma treatment and chemical methods such as alkali treatment (mercerization), silane treatment, and graft copolymerization have been investigated in this field. In particular, surface treatment by the silane coupling agent is used widely for various purposes [30,31]. Over the last two decades, various investigations have been conducted on the effects of the surface treatment on the mechanical properties of these biomass composites [3,4,9,29,30,32,33]. These studies have demonstrated that the suitable surface treatment of natural fiber improves the mechanical properties of these biomass composites.

However, to date, little interest has been paid to the surface treatment of natural fibers in polymeric tribomaterials based on such composites. It is well known that the surface treatment of natural fibers improves the interface adhesion between natural fibers and matrix polymer as mentioned earlier [3,4,13,20,21,22,29,30,32,33]. As a result, this improvement of the interface adhesion not only improves mechanical properties but also tribological properties such as frictional and wear properties of such composites. In other words, this interfacial adhesion plays a substantial role in controlling the tribological properties of these biomass composites [20]. Few studies have been reported on the effect of the surface treatment of fiber on the tribological properties of such composites [13,16,20,21,22,34,35,36,37,38,39]. Most of these studies have investigated the effects of alkali treatment using sodium hydroxide (NaOH) solution. These studies also report the results of the research on oil palm fiber-reinforced polyester composites by Yousif et al. [16,20], that of betelnut fiber-reinforced polyester composites by Nirmal et al. [21,34], that of sugar palm fiber-filled phenolic composites by Rashid [22] etc. In other studies by Chand and Dwivedi, the influence of the coupling agent, maleic anhydride grafted polypropylene (PP-g-MA), in this case on the abrasive wear behavior of chopped jute [35] and sisal fiber [36] polypropylene composites, was investigated. Chand and Dwivedi [37] also studied the effects of the surface treatment of fiber by the silane coupling agent, which was directly added to polyester resin, on the tribological properties of sisal fiber-reinforced polyester composites. On the other hand, Siva et al. [38] applied the silane coupling agent to the fiber as pre-treatment before for the surface treatment of coconut fiber in polyester composites. Moreover, Goriparthi et al. studied the effects of the type of various surface treatments, including two types of silane coupling agent used for pre-treatment, on the abrasive wear performance of jute fiber-reinforced PLA biomass composites [39]. These results showed that the tribological properties of these surface-treated natural fiber-reinforced polymer composites significantly improved as compared to those of the untreated ones. In particular, the most effective method for controlling interface adhesion between natural fiber and matrix polymer in these biomass composites was found to be the combination of alkali treatment and silane coupling agent. This is because proper surface treatment of natural fiber by alkali treatment and silane coupling agent can increase the interfacial adhesion between natural fiber and matrix polymer, and improve the mechanical and tribological properties. However, there are only few published studies on the influence of the surface treatment, other than alkali treatment by NaOH, on the tribological properties of these biomass composites [20,21].

In previous studies, we investigated the thermal, rheological, mechanical and tribological properties of hemp fiber (HF) reinforced plant-derived polyamide 1010 (PA1010) biomass composites (HF/PA1010) to develop new engineering materials and tribomaterials made of 100% inedible plant-derived materials [7,40,41,42,43,44,45,46,47]. Hemp fiber is a bast fiber crop and an annual plant that grows in temperate climates [4]. The surface of HF is pre-treated by alkali treatment by NaOH and by aminosilane coupling agent (3-(2-aminoethylamino) propyltrimethoxy silane, A-1120). On the other hand, PA1010 is made from sebacic acid and decamethylenediamine, which are obtained from plant-derived castor oil [48]. As castor oil is not used for food, there is no competition with human food production. It was found that the addition of HF, the surface treatment of HF such as alkali treatment by NaOH, and the surface treatment by aminosilane coupling agent, have strong influences on the thermal, rheological, mechanical and tribological properties of HF/PA1010 biomass composites [7,40,41,42,43,44,45,46,47]. In particular, the effect of surface treatment on these properties of HF/PA1010 biomass composites differs according to whether alkali treatment by NaOH is performed on the aminosilane coupling agent uses or not [8,40,45,47]. However, to further enhance the mechanical and tribological properties of all plant-derived polymer-based biomass composites, it is necessary to clarify of the effects of the type of silane coupling agent and volume fraction of natural fiber such as HF on the tribological properties of these biomass composites.

To improve the performance of all inedible plant-derived materials for new polymeric tribomaterials, this study aimed to experimentally investigate the effects of silane coupling agent on the tribological properties, namely the frictional coefficient, specific wear rate, and limiting *pv* (pressure *p* × velocity *v*) values, of hemp fiber-reinforced plant-derived polyamide 1010 biomass composites. In this study, three types of silane coupling agents such as aminosilane, epoxysilane and ureidosilane were used for the surface treatment of hemp fibers.

## 2. Materials and Methods

### 2.1. Materials

The materials used in this study were various surface-treated hemp fiber-reinforced plant-derived polyamide 1010 biomass composites [7]. Plant-derived polyamide 1010 (PA1010, Vestamid Terra DS16, Daicel Evonic Ltd., Tokyo, Japan) was used as the matrix polymer. Hemp fiber (HF, *φ*50–100 μm, Hemp Levo Inc., Tokyo, Japan) was used as a reinforcement fiber. HF was precut into 5 mm pieces and surfaced-treated by two types of surface treatment: (a) alkali treatment by sodium hydroxide (NaOH) solution and (b) surface treatment by various silane coupling agents. Alkali treatment by NaOH was employed as follows: 5% NaOH solution was placed in a stainless beaker. Chopped HFs were then added to the beaker and stirred well. This was kept at room temperature for 4 h. The fibers were then washed thoroughly with water to remove the excess NaOH sticking to the fibers. The alkali-treated fibers were dried in the air for 24 h and in a vacuum oven at 80 °C for 5 h. Three types of silane coupling agents, namely aminosilane (S1, 3-(2-aminoethylamino) propyltrimethoxy silane, A-1120, Momentive Performance Materials Inc., Waterford, NY, USA), epoxysilane (S2, 3-glycidoxypropyltrimethoxy silane, A-187), and ureidosilane (S3, 3-ureidopropyltrimethoxy silane, A-1160) were used as the surface treatment agents. The code, functional group, grade, chemical name and chemical structure of these three silane coupling agents are listed in Table 1. The treatment of HFs with the concentration of 1 wt % of the chosen silane coupling agents was carried out in deionized water (for S1) or 0.5 wt % acetic acid aqueous solution (for S2 and S3, where the pH of the solution was adjusted to 3.5) and stirred continuously for 15 min. Then, the fibers were immersed in the solution for 60 min. After treatment, fibers were removed from the solution and dried in air for 24 h and in a vacuum oven at 80 °C for 5 h. The volume fraction of fiber *V_f_* was fixed with 20 vol % in this study. To investigate the influence of *V_f_* on the tribological properties of HF/PA1010 biomass composites, ureidosilane (S3) treated HF/PA1010 biomass composites with the HF content of 10 vol %, 20 vol % and 30 vol % were prepared. The volume fraction of hemp fiber *V_f_* in composites was estimated by using the following relationship:

(1) $$V_{f}=\left[\left(\rho_{c}-\rho_{m}\right)/\left(\rho_{f}-\rho_{m}\right)\right]\times 100$$

where *V_f_* is the volume fraction of fiber, *ρ_c_* is the specific gravity of composites, *ρ_m_* is the specific gravity of the matrix, and *ρ_f_* is the specific gravity of fiber.

### 2.2. Processing

Various surface-treated HF/PA1010 biomass composites were extruded using a twin screw extruder and injection-molded [7,42]. All the components were dried for 12 h at 80 °C in a vacuum oven beforehand until the moisture level was below 0.2%, then dry blended in a small bottle, and subsequently the melt was mixed at 85 rpm and 220 °C using a twin screw extruder (TEX-30HSS, Japan Steel Works, Ltd., Tokyo, Japan). After mixing, the extruded strands of various HF/PA1010 biomass composites were cut into 5 mm pieces using a pelletizer and dried again at 24 h at 80 °C in a vacuum oven. Various shaped samples for various experiments were injection molded (NS20-A, Nissei Plastic Industrial, Nagano, Japan). The molding conditions were as follows: cylinder temperatures of 220 °C, mold (cavity) temperature of 30 °C, and the injection rate of 13 cm^3^/s. At this processing (mixing or injection) temperature of 220 °C, the dehydration of HF as well as the thermal degradation of lignin and hemicellulose occur slightly [7]. To maintain the dry conditions of the specimens in all the measurements, they were kept in accordance with JIS K 6920-2 for at least 24 h at 23 °C in desiccators after molding [49]. The details of composites used in this study are listed in Table 2. Reinforced composites of untreated HF, which was not surface-treated by alkali treatment and silane coupling agent, were used to compare the effect of surface treatment by silane coupling agent on the tribological properties of these biomass composites in this study.

### 2.3. Mechanical Properties

Three kinds of mechanical properties were measured: tensile, impact and hardness characteristics [49,50]. Tensile tests (number of samples *n* = 5) were carried out with dog-bone samples (12 mm × 60 mm × 2 mm, length of parallel part of 20 mm) on a universal tester (Strograph V-10, Toyo Seiki Seisaku-sho, Ltd., Tokyo, Japan), and performed at room temperature in accordance with JIS K 7113, and at the crosshead speed of 2 mm/min. Tensile strength, tensile modulus and elongation at break were obtained from stress-strain curves. Impact characteristics were determined by the Izod impact test. Izod impact tests (number of samples *n* = 10) were conducted using coupon specimens (12.7 mm × 64 mm × 3 mm) on a digital impact tester (DG-IB, Toyo Seiki Seisaku-sho, Ltd., Tokyo, Japan) at room temperature in accordance with JIS K 7110. The specimens were machined with a notch depth of 2.54 mm. Hardness characteristics were determined by type *D* of the durometer hardness (*HDD*) test. The durometer hardness test was conducted using plate specimens (30 mm × 30 mm × 3 mm) on a digital hardness testing machine (ASTM D) at room temperature, in accordance with JIS K 7215 (number of samples *n* = 10). In addition, to understand the structure of these biomass polymer composites, the surface of the samples fractured cryogenically in liquid nitrogen was observed using a scanning electron microscope (SEM, JSM6360LA, JEOL Ltd., Tokyo, Japan). The surfaces were sputter-coated with platinum (Pt).

### 2.4. Tribological Properties

The tribological properties were measured using a ring-on-plate type sliding wear tester (EFM-3-EN, Orientec, Co. Ltd., Tokyo, Japan) [49,50]. The tests were conducted using plate specimens (30 mm × 30 mm × 3 mm) with the surface (*R_a_* = 0.4 μm) finished by No. 240 and No. 800 polishing paper at room temperature under dry conditions in accordance with JIS K 7218a (tested by full contact between the test specimen and metal counterface). A carbon steel (S45C) ring (*φ*20 mm × *φ*25.6 mm × 15 mm, apparent contact area *A_a_* = 2 cm^2^) with the surface (*R_a_* = 0.4 μm) finished with the same polishing paper was used as a metal counterpart. A thermocouple, which was attached at a height of 1 mm from the lower part of the counterface, was used to estimate for the apparent frictional surface temperature *T* during the sliding wear test. In this study, two types of tribological test were carried out: the constant normal load and constant sliding velocity test, and the limiting *pv* (pressure *p* × velocity *v*) value test by the step load method.

First, the sliding wear test (number of samples *n* = 3) under constant normal and constant sliding velocity was conducted at the normal load *P* of 50 N (apparent contact pressure *p_a_* = 0.25 MPa), the sliding velocity *v* of 0.2 m/s, and the sliding distance *L* of 600 m. Tribological properties were evaluated by the kinetic frictional coefficient *μ* and specific wear rate *V_s_*. The kinetic frictional coefficient was calculated by the average value from 400 m to 600 m, which is the stable (steady state). The specific wear rate was calculated by the weight of the sample (plate) before and after test from the following equation:

*V_s_* = *V*/*PL* (2)

where *V* is the volume loss, *P* is the normal load and *L* is the sliding distance. The volume loss *V* is given by the mass loss *M* of polymeric specimens divided by the specific gravity of the composite material *ρ*. The specific gravity *ρ* was measured by the electronic balances (EB-60S, Shimadzu, Co., Kyoto, Japan) at room temperature in accordance with JIS K 7112a (the water displacement method). The sliding surfaces on the test specimen, wear debris, and counterface before and after tests, were observed using a SEM with platinum (Pt) sputter coated.

Second, the limiting *pv* (pressure *p* × velocity *v*) value test by the step load method (number of samples *n* = 3) was conducted under a constant velocity *v* of 0.3, 0.4 and 0.5 m/s at the initial normal load *P*_0_ of 50 N (apparent contact pressure *p_a_* = 0.25 MPa) and step load *P* of 25 N every 3 min until the test piece fractured or melted. The step load just before the test piece fractured or melted was defined as the limiting load *P_lim_*. This limiting load was divided with the apparent contact area *A_a_* = 2 cm^2^, and the value obtained was taken as the apparent limiting contact pressure *p*. The limiting *pv* value was calculated by multiplying this limiting contact pressure and the test velocity *v*.

## 3. Results and Discussion

### 3.1. Material Characterization

Characterization such as the mechanical properties and the morphology of materials used in this study are discussed in this section before the tribological testing. First, the mechanical properties of various surface-treated hemp fiber-reinforced plant-derived polyamide 1010 biomass composites (HF/PA1010) are discussed. Mechanical properties such as tensile, Izod impact and durometer hardness characteristics of various surface-treated HF/PA1010 biomass composites are listed in Table 3. Tensile strength *σ_t_*, tensile modulus *E_t_* and durometer hardness *HDD* of various surface-treated HF/PA1010 biomass composites increase with increasing filling HF, volume fraction of fiber *V_f_*, and with their surface-treatment using both alkali treatment (NaOH) and various silane coupling agents, although elongation at break *ε_t_* and Izod impact strength *a_iN_* decreased. In particular, the improvement effect of filling HF and *V_f_* on the mechanical properties of those biomass composites is much higher than that of fiber surface-treatment by various silane coupling agents. Moreover, the difference between the mechanical properties of HF-S3 with 10 vol % and that with 20 vol % is low. This behavior may be due to factors such as changes in the fiber dispersion of the samples, and the high improvement effect of filling HF, however it is difficult to establish the reasons. On the other hand, the influence of the type of silane coupling agent on the mechanical properties is slight, however the degree of influence differs according to the type of silane coupling agent. In addition, *σ_t_* and *HDD* of various surface-treated HF/PA1010 biomass composites increase in the following order: HF (untreated) = HF-S2 (epoxysilane) < HF-S1 (aminosilane) < HF-S3 (ureidosilane). In other words, it should be noted that surface-treatment by ureidosilane coupling agent (S3) is the most effective silane system for the enhancement of mechanical properties such as strength and hardness. These may be attributed to the change of interfacial interaction between fiber (HF) and matrix polymer (PA1010) according to the type of silane coupling agent used. The interfacial adhesion between HF and PA1010 is thought to improve with surface treatment by both alkali treatment (NaOH) and various silane coupling agents. It is known that the *HDD* of natural fiber-reinforced polymer composites is softer than that of glass fiber-reinforced ones [51]. This has the advantage of reducing the wear of the extruder screw. However, it is necessary to investigate these in future comparative studies.

**Figure 1:**
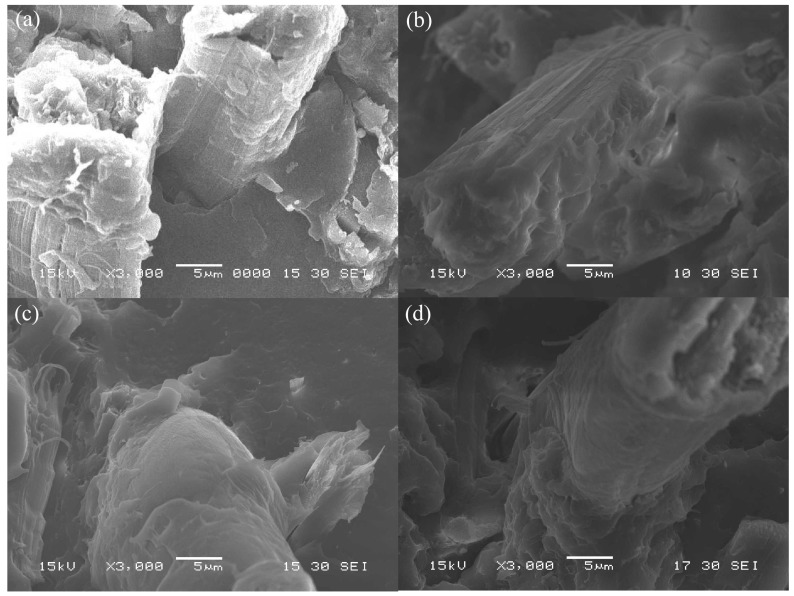
SEM photographs of fracture surface for various surface-treated HF/PA1010 biomass composites: (**a**) HF; (**b**) HF-S1; (**c**) HF-S2 and (**d**) HF-S3 (×3000).

To clarify the internal microstructures of various surface-treated HF/PA1010 biomass composites such as the interfacial interaction between fiber (HF) and matrix polymer (PA1010), we discuss the morphologies of these various surface-treated HF/PA1010 biomass composites. We performed the SEM observation of the fractured surface cryogenically in liquid nitrogen. Figure 1 shows the representative SEM photographs of the cryogenically fractured surface of various surface-treated HF/PA1010 biomass composites: HF (untreated) (Figure 1a), HF-S1 (aminosilane) (Figure 1b), HF-S2 (epoxysilane) (Figure 1c), and HF-S3 (ureidosilane) (Figure 1d), respectively. Observation of these SEM images shows differences in the interfacial interaction between HF and PA1010. The morphology of HF (untreated, Figure 1a) indicates that the fractured surface with fiber had been pulled out, with a relatively clean fiber surface and without matrix polymer, and some gaps are present at the interface between HF and PA1010. These findings indicate poor chemical and physical contact between HF and PA1010. On the other hand, the morphologies of biomass composites surface-treated by both alkali treatment (NaOH) and various silane coupling agents (Figure 1b–d) indicate a fracture surface with coating matrix polymers on the fiber surface, and the fiber does not leave any voids at the interface between HF and PA1010. Thus, these findings confirm good interfacial interaction between the fiber and matrix polymer. This may be attributed to the chemical reaction between the possible reaction site on the HF by NaOH alkali treatment and the various functional groups in the silane coupling agent. In our previous studies [7,47], we carried out chemical analysis such as Fourier transform infrared spectroscopy (FT–IR) in order to determine the chemical composition of the surface of HF with various surface treatments. The characterization of the fiber surface using FT–IR demonstrated that the alkali treatment by NaOH is able to remove lignin, wax, and hemicellulose from fiber bundles and replace more OH groups on the HF surfaces. In addition, the surface-treatment by various silane coupling agents adds peaks associated with the possible chemical reaction sites on the HF. Incidentally, Sigriccia et al. [52] reported similar results on the removal of the lignin and hemicellulose, and the presence of silane in hemp fiber using FT–IR and SEM observation. Accordingly, these SEM image observations show the composition of the surface of HF with various surface treatments using FT–IR and the mechanical properties for the enhancement of rigidity as mentioned earlier.

### 3.2. Tribological Properties by the Constant Normal Load and Constant Sliding Velocity Test

**Figure 2:**
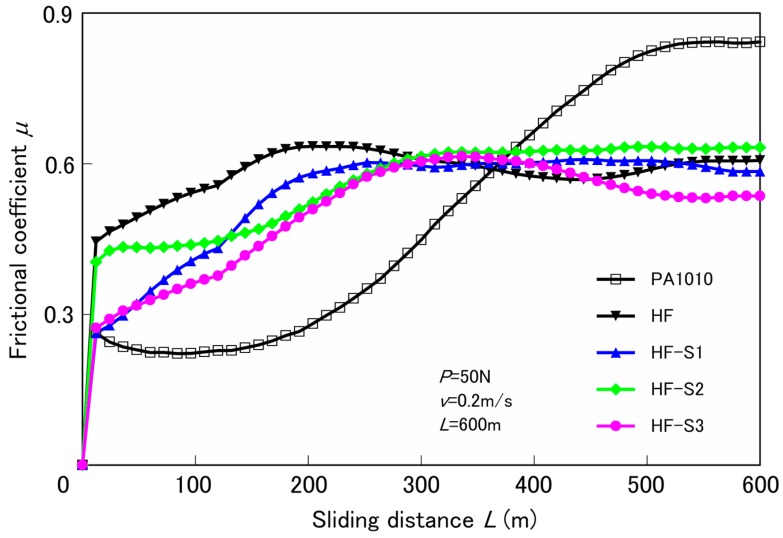
Frictional coefficient as a function of sliding distance for various surface-treated HF/PA1010 biomass composites.

Tribological properties using a ring-on-plate type sliding wear tester by constant normal load and constant sliding velocity under dry conditions are discussed below. Figure 2 shows the typical relationship between the frictional coefficient *μ* and sliding distance *L* (*μ*-*L* curves) of various surface-treated HF/PA1010 biomass composites against carbon steel (S45C) ring at the constant normal load *P* of 50 N, constant sliding velocity *v* of 0.2 m/s, and sliding distance *L* of 600 m. The *μ*-*L* curves of pure plant-derived PA1010 (100%) and various surface-treated HF/PA1010 biomass composites showed different behaviors according to the type of materials. The *μ* of pure PA1010 increases about 0.3 immediately and stabilizes up to 200 m, and then *μ* gradually increases up to about 0.8 from *L* = 200 m to 400 m, after which the value becomes more or less stable after *L* = 400 m. In short, the running-in period of pure PA1010 is long by 400 m, and then shifts to the steady-state period. This may be attributed to the increase in the real contact area between the pure PA1010 sample and counterface of the carbon steel (S45C) ring caused by the softening of the polymeric sample surface. This is because the temperature of the polymeric sample surface gradually increases with the frictional heat generated during the ring-on-plate type sliding wear test, in which the type of full contact between the test specimen and metal counterface is tested. The results indicate that not only does the frictional coefficient increase, but also the wear of polymeric sample progresses abruptly. On the other hand, the *μ*-*L* curves of various HF/PA1010 biomass composites demonstrate different behaviors. The *μ* of these biomass composites increases abruptly soon after the initial run, then gradually rises up to about 0.6 at *L* = 300 m, after which it stabilizes with or without surface-treatment by various silane coupling agents. To summarize, increasing the *μ* region associated with the initial running-in period of HF/PA1010 biomass composites is smaller than that of pure PA1010. This is probably due to the change in the mode of friction mechanism when filled with HF. In other words, the contact areas between the polymer and the metallic counterface are reduced by introducing discontinuous phases [37]. It also has a load-carrying capacity effect caused by fiber filling. However, the *μ*-*L* curves shift to the steady-state period slightly differently for each type of surface treatment.

**Figure 3:**
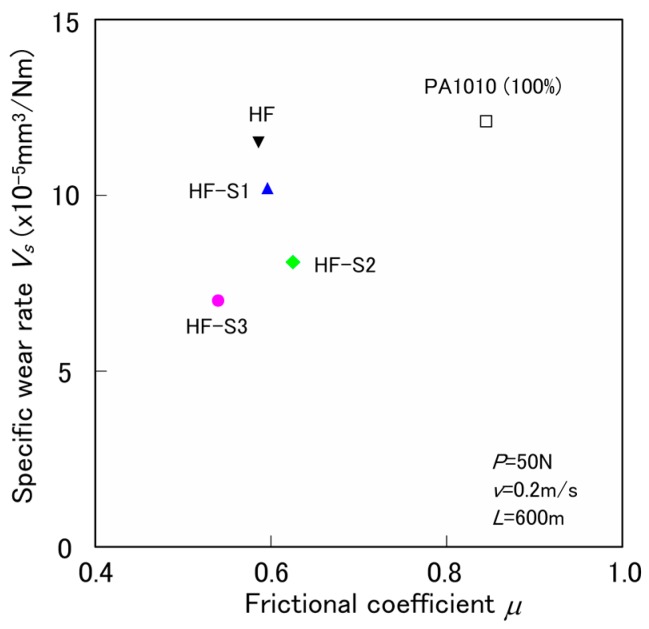
Tribological properties (*P* = 50 N, *v* = 0.2 m/s, and *L* = 600 m) of various surface-treated HF/PA1010 biomass composites.

Figure 3 shows the relationship between the specific wear rate *V_s_* and the frictional coefficient *μ*, of various surface-treated HF/PA1010 biomass composites by constant normal load *P* of 50 N, constant sliding velocity *v* of 0.2 m/s, and sliding distance *L* of 600 m. The *V_s_* was calculated by the mass loss of the polymer composites plate before and after testing, and the *μ* was calculated by the average value from 400 m to 600 m in Figure 2, which is the steady-state period. The *μ* and *V_s_* of pure plant-derived PA1010 (100%) improved when filled with HF and surface-treated by the combination of NaOH alkali treatment and various silane coupling agents. However, the effect of the filling of HF on the degree of reduction in *μ* was higher than that of the type of surface-treatment by the silane coupling agent. The *μ* of various surface-treated composites decreases in the following order: PA1010 >> HF-S2 (epoxysilane) > HF-S1 (aminosilane) > HF (untreated) > HF-S3 (ureidosilane). On the other hand, the *V_s_* of the composites shows different behavior from that of *μ*, decreasing in the following order: PA1010 > HF > HF-S1 > HF-S2 > HF-S3. Unlike *μ*, the effect of the type of surface-treatment by the silane coupling agent is higher than that of the filling of HF. Notably, S3 (ureidosilane coupling agent) showed the best improvement effect on the tribological properties, both *μ* and *V_s_*, of HF/PA1010 biomass composites. These results may be attributed to the change in the mode of friction and wear mechanism by the type of silane coupling agent caused by the good interaction and interphase adhesion between the hemp fiber and the matrix polymer of plant-derived PA1010 and good dispersion of fiber in the composites.

**Figure 4:**
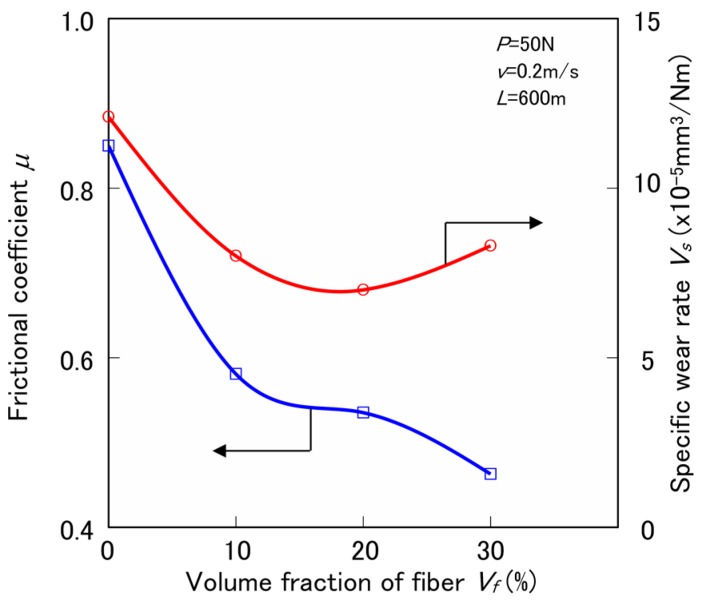
Tribological properties of various surface-treated HF/PA1010 biomass composites with ureidosilane coupling agents (S3).

Figure 4 shows the influence of volume fraction of hemp fiber *V_f_* on the frictional coefficient *μ* and the specific wear rate *V_s_* of ureidosilane (S3) treated HF/PA1010 biomass composites (HF-S3/PA1010) by constant normal load *P* of 50 N, constant sliding velocity *v* of 0.2 m/s, and sliding distance *L* of 600 m. *μ* of HF-S3/PA1010 biomass composites decreases with increasing *V_f_* although *V_s_* of HF-S3/PA1010 biomass composites has a minimum peak at 20 vol %. In other words, the effect of *V_f_* on the tribological behavior of these HF/PA1010 biomass composites differs for each tribological property. These results may be due to the change in the mode of friction and wear mechanism by volume fraction of fiber *V_f_* caused by the fiber dispersion and orientation in the composites. The evidence on the mechanisms of these tribological behaviors will be discussed in the next section.

### 3.3. SEM Observation and Wear Mechanism

SEM observations were discussed in the order of metallic counterface, that of wear debris, that of worn surface of the materials, and wear mechanism. Tribological properties such as the frictional coefficient and specific wear rate are improved when filled with hemp fibers, or when surface treated by combinations of NaOH alkali treatment and various silane coupling agents, and by volume fraction of fiber. These results may be attributed to the change in the mode of friction and wear mechanism as a result of these factors. Therefore, it is necessary to observe the formation of transfer films on the metallic counterface, wear debris, and worn surface of the materials in order to understand the mechanisms of tribological behavior, because those of polymer and polymer composites are strongly influenced by their ability to form these [49,50].

#### 3.3.1. Metallic Counterface

**Figure 5:**
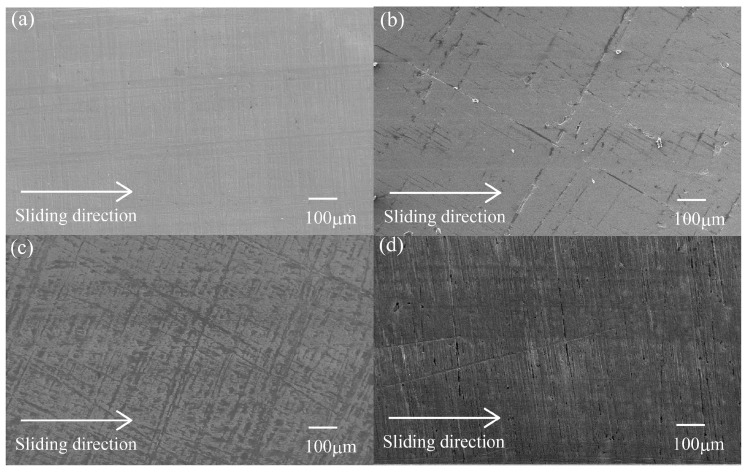

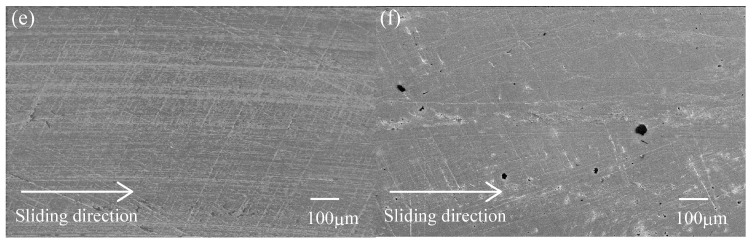
SEM photographs of the metallic counterface before and after sliding wear tests of various surface-treated HF/PA1010 biomass composites (*V_f_* = 20 vol %): (**a**) Before test; (**b**) PA1010; (**c**) HF; (**d**) HF-S1; (**e**) HF-S2 and (**f**) HF-S3.

Figure 5 presents the SEM photographs of the metallic counterface before and after sliding wear tests against various surface-treated HF/PA1010 biomass composites (*V_f_* = 20 vol %): before sliding wear test (Figure 5a), neat PA1010 (Figure 5b), HF (untreated, Figure 5c), HF-S1 (aminosilane, Figure 5d), HF-S2 (epoxysilane, Figure 5e) and HF-S3 (ureidosilane, Figure 5f), respectively. The counterface before the sliding wear test (Figure 5a) had a fine scratched mark on the surface caused by the constant surface roughness (*R_a_* = 0.4 μm) finished by polishing paper. These SEM observations of the counterface after the sliding wear test showed differences in the formation of transfer films on the metallic counterface according to the type of materials. The counterface after the sliding wear test against pure PA1010 (Figure 5b) showed a thick and grainy transfer film with scratch marks all over the surface. There was also a small amount of wear debris on the counterface. On the contrary, that of various HF/PA1010 biomass composites (Figure 5c–f) had thin small scratches all over the surface, which can often be seen in the constant surface roughness finish, although the thickness of the transfer films on the counterface differs for each type of surface-treatment. This thickness was also found to decrease in the following order: untreated HF (Figure 5c) > HF-S1 (Figure 5d) > HF-S2 (Figure 5e) > HF-S3 (Figure 5f). In particular, the thickness of transfer films on the counterface against HF-S3/PA1010 biomass composites (Figure 5f) was thinner than that of other surface-treated ones. There were also several large amounts of wear debris adhered on the counterface for HF-S3. It is well known that the tribological properties of polymer composites improve when smooth, thin and uniform transfer films are formed on the metallic counterface [49,50]. This is because friction repeatedly occurs between the polymer materials and transfer film instead of the metallic counterface, preventing the transfer film from dropping to the sliding surface.

#### 3.3.2. Wear Debris

**Figure 6:**
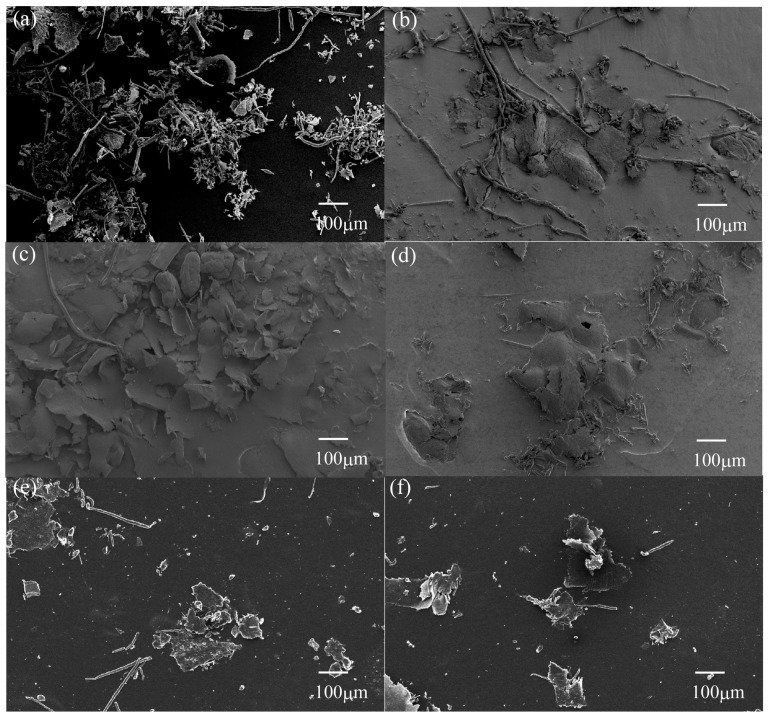
SEM photographs of wear debris after sliding wear tests of various surface-treated HF/PA1010 biomass composites: (**a**) PA1010; (**b**) HF; (**c**) HF-S1; (**d**) HF-S2; (**e**) HF-S3-20 and (**f**) HF-S3-30.

Figure 6 shows the SEM photographs of wear debris after sliding wear tests of various surface-treated HF/PA1010 biomass composites against the S45C ring: neat PA1010 (Figure 6a), HF (Figure 6b), HF-S1 (Figure 6c), HF-S2 (Figure 6d), HF-S3 (Figure 6e), and HF-S3-30 (Figure 6f), respectively. These examples of wear debris were collected from the outside of the sliding surface after the sliding wear test to determine the wear mechanism of various surface-treated HF/PA1010 biomass composites. The shape and size of the wear debris changed when filled with hemp fibers and the type of surface treatment.

Those of neat PA1010 (Figure 6a) were small and long filamentary (roll) particles; those of untreated HF ones (Figure 6b) were a mixture of big flaky and long filamentary particles; and those of cases surface treated by the combination of NaOH alkali treatment and various silane coupling agent (Figure 6c–f) were a mixture of many large flaky, and some long filamentary, particles. It is well known that the filamentary (roll) particles are often observed in various pure polyamides (PA) such as PA66 [53] and PA11 [54]. Because the intermolecular forces of PA are strong and the molecular orientation is low, PA does not flow easily, and the contact parts are pulled and cut [55]. On the other hand, the wear debris for filled polyamide composites had various shapes according to the type of fillers and fibers. In this study, the shape and size of wear debris of various HF/PA1010 biomass composites change from short and long filamentary (roll) particles to a mixture of some long filamentary and many big flaky ones. The long filamentary wear debris is thought to derive from PA1010. On the other hand, the big flaky wear debris is considered to be due to the filling HF and the surface treatment by the combination of NaOH alkali treatment and various silane coupling agents. In the case of various HF/PA1010 biomass composites, the small wear debris items are connected to each other in the presence of hemp fibers. As a result, these grow into the large flaky particles, and are thinly stretched on the sliding surfaces. In short, these observations may support the change in the mode of wear mechanism when filled with HF and surface-treated by the combination of NaOH alkali treatment and various silane coupling agents caused by good interfacial interaction between fiber and matrix polymer and good dispersion of fiber in the composites. However, the effect of type of surface treatment by various silane coupling agents on the shape and size of this wear debris could hardly be seen.

#### 3.3.3. Worn Surface

**Figure 7:**
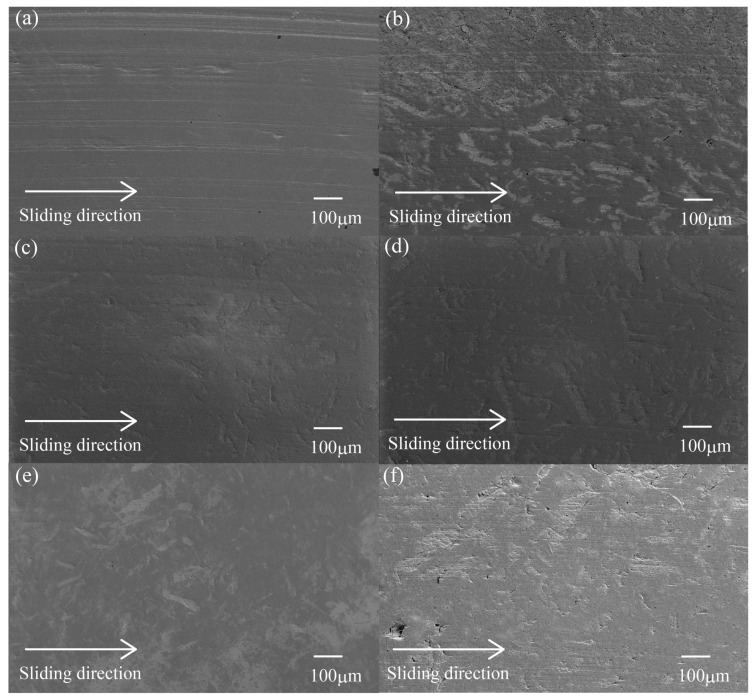
SEM photographs of the worn surface after sliding wear tests of various surface-treated HF/PA1010 biomass composites: (**a**) PA1010; (**b**) HF; (**c**) HF-S1; (**d**) HF-S2; (**e**) HF-S3-20 and (**f**) HF-S3-30.

Figure 7 demonstrates the SEM photographs of the worn surface after sliding wear tests against the metallic counterpart (S45C) of various surface-treated HF/PA1010 biomass composites: pure PA1010 (Figure 7a), HF (Figure 7b), HF-S1 (Figure 7c), HF-S2 (Figure 7d), HF-S3 (Figure 7e), and HF-S3-30 (Figure 7f), respectively. The morphologies of these worn surfaces are changed when filled with HF and the type of surface treatment by combination of NaOH alkali treatment and various silane coupling agents. Furthermore, the difference in the morphologies of the worn surface of various surface-treated HF/PA1010 biomass composites appears more conspicuously than the formation of transfer films on the metallic counterface and the shape of the wear debris, as mentioned earlier. The worn surface of pure PA1010 (Figure 7a) clearly shows the rough face caused by the softening and deformation of the matrix polymer, and there are countless deeply frictional marks in the direction parallel to the sliding direction. On the other hand, the worn surfaces of untreated HF/PA1010 biomass composites (Figure 7b) also show rough and bumpy faces, and have some frictional marks. Moreover, many hemp fibers are clearly seen on the worn surface, and these fibers are slightly debonded in the gap between the fiber and matrix polymer, and tear off. In contrast, the morphologies of the worn surface of various surface-treated HF/PA1010 biomass composites (Figure 7c–e) with 20 vol % hemp fiber content systems have a much smoother face than that of untreated HF/PA1010 biomass composites (Figure 7b). Those of HF/PA1010 biomass composites differ according to the type of silane coupling agent, although the frictional marks of those systems are very shallow. In addition, hemp fibers could not be clearly seen on the worn surfaces of surface-treated HF/PA1010 biomass composites compared to those of untreated HF ones. Furthermore, hemp fibers on the worn surface of surface-treated biomass composites (Figure 7c–e) were not severely damaged. In other words, fiber debonding, breakage, and the detachment of fibers on the worn surface do not occur due to good interfacial interaction between the fiber and matrix polymer.

**Figure 8:**
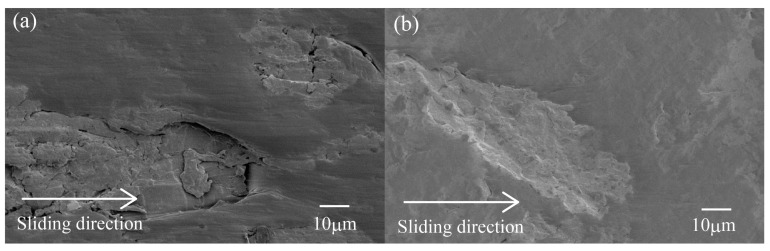
SEM photographs of the worn surface after sliding wear tests of various surface-treated HF/PA1010 biomass composites: (**a**) HF (Magnification of Figure 7b) and (**b**) HF-S3-20 (Magnification of Figure 7e).

For a better understanding of the friction and wear mechanism of HF/PA1010 biomass composites, Figure 8 shows the magnified view of the worn surface of a part of Figure 7 observed at high magnification of 1000. Here, Figure 8a was observed on the worn surface of untreated HF/PA1010 biomass composites (Figure 7b), and Figure 8b of ureidosilane-treated HF-S3/PA1010 biomass composites (Figure 7e), respectively. The worn surface of untreated HF (Figure 8a) shows fragments, fiber debonding and broken fibers during the sliding process. The untreated fiber regions are more abraded than the softening matrix polymer regions. This may be attributed to low mechanical properties such as shear and fracture caused by poor interfacial interaction between the fiber and matrix polymer. As a result, untreated HF/PA1010 biomass composites showed poor wear resistance. On the contrary, the worn surface of HF-S3 (Figure 8b) did not show fiber debonding with the matrix polymer. The degree of wear was, also, almost the same betweem the surfaces treated by ureidosilane in the S3 hemp fiber regions and matrix polymer regions. The differences in the morphologies of the worn surface for either untreated fibers or surface-treated filled composites have been reported by most works on other natural fiber-reinforced polymer composites [15,16,20,34,37,39]. These behaviors may be explained by the following mechanisms: first, the interfacial interaction between fiber and matrix polymer caused by the surface treatment by S3 (ureidosilane) is stronger than the frictional force, what is called a good interfacial interaction effect; second, the hemp fiber is reinforced with the surface treatment by a silane coupling agent such as S3 (ureidosilane), what is called the fiber protection effect; third, the back transfer films, which dropped from the couterface during the sliding process, cover the friction surface, what is called the back transfer film effect [15]. The differences in the worn surface depending on whether the silane coupling agent is used in the surface treatment changed according to the combination of these three effects. Although the detailed results of other types of silane coupling agent are not discussed here, their worn surface morphologies were more or less the same as those of the S3 (ureidosilane) treated composites.

Next, the worn surface of HF-S3-30 biomass composites (Figure 7f), which have a volume fraction of fiber at 30 vol %, has basically the same behavior as HF-S3 treated composites (20 vol %, Figure 7e). However, a part of the worn surface of Figure 7f also shows debonding and detachment of fibers. This may be attributed to the relative decrease in the amount of matrix polymer regions with the increasing volume fraction of fiber. The fiber dispersion in the composites was poor. Consequently, the back transfer film effect is partially reduced compared with 20 vol % HF-S3/PA1010 biomass composites. The wear resistance of 30 vol % HF-S3/PA1010 biomass composites becomes lower than that of 20 vol % ones.

#### 3.3.4. Discussion

SEM observations of the metallic counterface, wear debris, and worn surface of various surface-treated HF/PA1010 biomass composites suggest the following wear mechanisms for the improvement of the tribological properties of these biomass composites: HF filling and the surface treatment effect by the combination of alkali treatment and silane coupling agent resulted in the formation of a thin, smooth and uniform transfer film on the metallic counterface. In addition, the HF filling effect had a stronger influence on the formation of the transfer film than the surface treatment effect by a combination of alkali treatment and silane coupling agent. The formation of the thin, smooth and uniform transfer film on the metallic counterface helps decrease friction and wear loss. Because friction repeatedly occurs between the polymer materials and transfer film instead of the counterface, this prevents the transfer film from dropping to the sliding surface. The shape and size of wear debris of various HF/PA1010 biomass composites have the same tendency as the formation of transfer films. When filled with HF and surface-treated by the combination of NaOH alkali treatment and various coupling agents, the shape and size of wear debris of various HF/PA1010 biomass composites change from short and long filamentary (roll) particles to a mixture of some long filamentary and many large flaky ones. That is, the HF filling effect has a stronger influence on the shape and size of wear debris than the surface treatment effect by a combination of alkali treatment and silane coupling agent. By contrast, the differences in the morphology of the worn surface of various surface-treated HF/PA1010 biomass composites appeared more conspicuous than the formation of transfer films and the shape and size of wear debris. In particular, the effect of surface treatment of the combination of NaOH alkali treatment and silane coupling agent and volume fraction of fiber influenced the wear mechanism remarkably. When surface-treated by the combination of NaOH alkali treatment and silane coupling agent, fiber debonding, breakage, and detachment of fibers on the worn surface under sliding process do not occur. This is caused by good interfacial interaction between the fiber and matrix polymer with these surface treatments. The detail of the wear mechanism with or without surface treatment of fiber was discussed in the previous section. On the other hand, the effect of volume fraction of fiber on wear loss has a minimum peak at 20 vol %. This may be attributed to the difference of fiber dispersion in the composites according to the volume fraction of fiber. Accordingly, the filling of hemp fiber, surface treatment by the combination of NaOH alkali treatment and various silane coupling agents, and volume fraction of fiber, have significant influence on the improvement of tribological properties of plant-derived PA1010. In particular, surface treatment by the combination of NaOH alkali treatment and silane coupling agent remarkably improved wear properties.

### 3.4. Limitting pv Value

**Figure 9:**
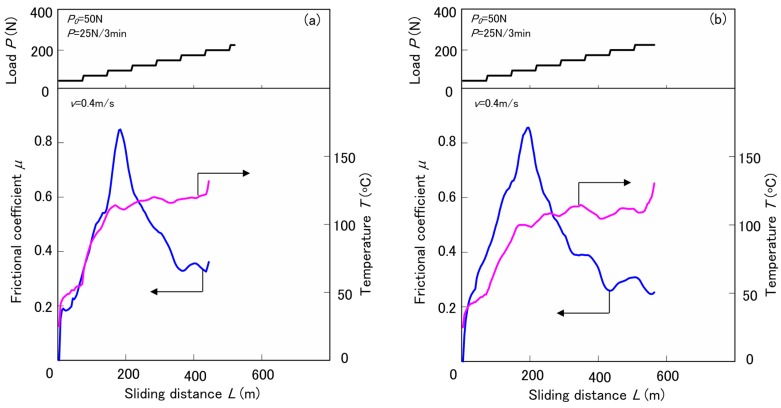
Frictional coefficient as a function of sliding distance measured by the step load test at *v* = 0.4 m/s for various surface-treated HF/PA1010 biomass composites: (**a**) HF and (**b**) HF-S3.

The limiting *pv* (pressure *p* × velocity *v*) values test results by the step load method of various surface-treated HF/PA1010 biomass composites, which is harsher than the constant load and speed test, are discussed in this section. These limiting *pv* values are used as the criteria to evaluate the critical operating conditions under which the materials fail, and to characterize the thermal and wear resistance of polymeric tribomaterials [56,57,58]. Figure 9 shows typical results, such as the frictional coefficient *μ* and apparent frictional surface temperature *T* as a function of sliding distance *L*, by the step load testing of various HF/PA1010 biomass composites: untreated HF (Figure 9a) and ureidesilane treated HF-S3 (Figure 9b) at sliding velocity *v* of 0.4 m/s, initial normal load *P*_0_ of 50N and step load *P* of 25 N/3 min. *μ* of both untreated HF and treated HF-S3 increases with increasing *L* up to about 200 m (3 step, that is 75 N), and then gradually decreases with increasing *L*. On the other hand, the *T* of both untreated HF and treated HF-S3 increases with *L*, however, the *T*-*L* curves do not increase uniformly. That is to say, *T* increases gradually with *L* up to about 110 °C and then becomes constant although *T* temporarily stagnates at about 50 °C. The former temperature is the equilibrium temperature *T_e_* measured by the step load method in this study, and the latter is the glass transition temperature *T_g_* of pure PA1010 [7]. The real temperatures on the frictional surface are expected to be higher than *T_e_*. If the *pv* value exceeds a critical value, a sharp temperature increase occurs after a certain time lapse, leading to melting, burning, degradation or failure. The minimum *pv* and the temperature at which these phenomena occur is defined as the limiting *pv* value and the limiting temperature *T_max_*, respectively [57]. *T_e_* of treated HF-S3 is the same as that of untreated HF although the distance (time) to reach *T_e_* of treated HF-S3 is longer than that of untreated HF. Thus the limiting *pv* value, which is the criteria for thermal and wear resistance, of HF/PA1010 biomass composites improves with surface treatment by the silane coupling agent.

**Figure 10:**
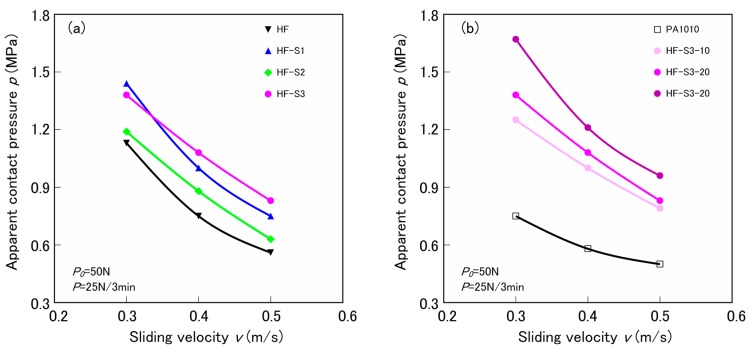
Apparent contact pressure as a function of sliding velocity for various surface-treated HF/PA1010 biomass composites: (**a**) Influence of type of silane coupling agent and (**b**) Influence of volume fraction of HF.

The apparent contact pressure *p* is plotted against the sliding velocity *v* in Figure 10 as *pv* curves to describe the effect of the type of silane coupling agent (Figure 10a) and the effect of the volume fraction of fiber (Figure 10b), respectively. Here, the apparent contact pressure is given by the limiting load *P_lim_*. This is the step load just before the test piece fractured or melted, divided by the apparent contact area *A_a_* = 2 cm^2^. As *p* of all materials increases with decreasing *v*, a slope of *pv* curves against *v* differs according to the type of silane coupling agent and the volume fraction of fiber *V_f_*. The effect of the type of silane coupling agent on the slope of *pv* curves of various surface-treated HF/PA1010 biomass composites (Figure 10a) increases in the following order: HF (untreated) < HF-S2 (epoxysilane) < HF-S1 (aminosilane) < HF-S3 (ureidosilane). By contrast, the influence of volume fraction of fiber on the slope of *pv* curves of surface-treated by ureidosilane HF-S3/PA1010 biomass composites (Figure 10b) increases with increasing *V_f_*.

**Figure 11:**
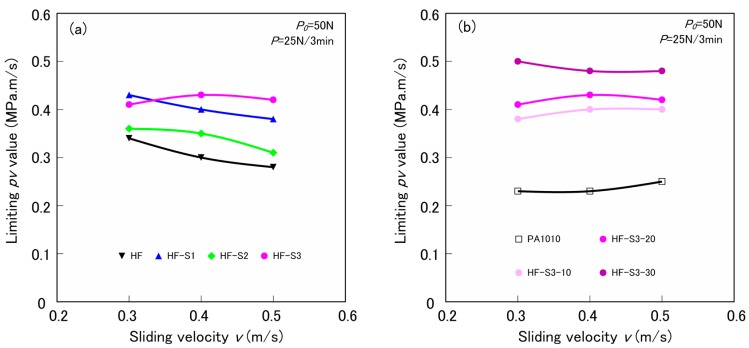
Limiting *pv* value as a function of sliding velocity *v* for various surface-treated HF/PA1010 biomass composites: (**a**) Influence of type of silane coupling agent and (**b**) Influence of volume fraction of HF.

Figure 11 shows the relationships between the limiting *pv* value and the sliding velocity *v* of various surface-treated HF/PA1010 biomass composites: the effect of the type of silane coupling agent (Figure 11a), and the effect of volume fraction of fiber (Figure 11b), respectively. Here, the limiting *pv* value is calculated by multiplying this apparent contact pressure *p* and the sliding velocity *v*. The limiting *pv* value has the same tendencies as those of *pv* curves, that is the effect of the type of the silane coupling agent and the volume fraction of fiber. However, the influence of sliding velocity on the limiting *pv* value of various surface-treated HF/PA1010 biomass composites can hardly be seen. These tendencies are similar to the mechanical properties of various surface-treated HF/PA1010 biomass composites shown in Table 3. This indicates that *pv* values are closely related to the load carrying ability. These results suggest the following mechanisms for improving the limiting *pv* value of various surface-treated HF/PA1010 biomass composites: the ability to support the load increases when filled with HF, the surface treatment by the combination of NaOH alkali treatment and silane coupling agent, and the volume fraction of fiber. The difference in the wear mode such as the formation of transfer film on the metal counterface, the wear debris, and the worn surface of the materials, are thought to change the limiting *pv* values with these factors. These results suggest that the surface treatment by ureido silane coupling agent (S3) is the most effective treatment method for improving the limiting *pv* values of these HF/PA1010 biomass composites. These effects increase with increasing volume of fraction fiber.

## 4. Conclusions

We studied the effects of silane coupling agent on the tribological properties of hemp fiber reinforced plant-derived polyamide 1010 biomass composites. The following results were obtained: 

Tribological properties by the constant normal load and constant sliding velocity test for HF/PA1010 biomass composites improved with the surface treatment by the silane coupling agent. In particular, the surface treatment effect on the specific wear rate of HF/PA1010 biomass composites was better than that on the frictional coefficient. These improvements may be attributed to the change in the mode of friction and wear mechanism by the type of silane coupling agent caused by the interaction and interphase adhesion between the hemp fiber and the matrix polymer of plant-derived PA1010. The influence of volume fraction on the tribological properties of ureidosilane treated HF/PA1010 biomass composites differed for each tribological property. This may be due to the change in the mode of wear mechanism by fiber dispersion and orientation in the composites.

In the SEM observations after the sliding wear test, the differences in the morphology of the worn surface of treated HF/PA1010 biomass composites were more conspicuous than the formation of transfer films and the shape and size of wear debris. In particular, the effects of surface treatment and volume fraction of fiber were found to significantly influence the wear mechanism. Surface treatment by silane coupling agent prevents the debonding, breakage, and detachment of fibers on the worn surface.

The limiting *pv* value improved when filled with hemp fiber, surface-treated by silane coupling agent, and increased volume fraction of fiber. These tendencies are similar to the mechanical properties of various surface-treated HF/PA1010 biomass composites in this study. In particular, the ureidosilane coupling agent (S3, A-1160) had the best improvement effect for tribological properties such as frictional coefficient, specific wear rate, and limiting *pv* value of HF/PA1010 biomass composites in this study.

However, there are very few studies on the comparisons of the tribological properties of hemp fiber (natural fiber) reinforced PA1010 biomass composites and those of glass fiber-reinforced PA1010 composites. Since this study focused on the effect of the silane coupling agent on the tribological properties of HF/PA1010 biomass composites, it will be necessary to conduct these comparative studies in the future.

## Figures and Tables

**Table 1:** Code, functional group, grade and chemical name of silane coupling agents used in this study.

Code	Functional Group	Grade	Chemical name	Structure
S1	Amino	A-1120	N-(2-aminoethyl)-3-aminopropyltrimethoxy silane	H_2_NCH_2_CH_2_NHCH_2_CH_2_CH_2_Si(OCH_3_)_3_
S2	Epoxy	A-187	3-glycidoxypropyltrimethoxy silane	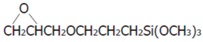
S3	Ureido	A-1160	3-ureidopropyltrimethoxy silane	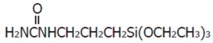

**Table 2:** Code, alkali treatment, silane coupling agent and volume fraction of fiber of HF/PA1010 biomass composites used in this study.

Code	Alkali Treatment	Silane Coupling Agent	Volume Fraction of Fiber *V_f_* (vol %)
PA1010	-	-	-
HF	-	-	20
HF-S1	NaOH	S1 (Aminosilane)	20
HF-S2	NaOH	S2 (Epoxysilane)	20
HF-S3	NaOH	S3 (Ureidosilane)	10, 20, 30

**Table 3:** Mechanical properties of various surface-treated HF/PA1010 biomass composites with silane coupling agents.

Code	*V_f_* (vol %)	Tensile Strength *σ_t_* (MPa)	Tensile Modulus *E_t_* (GPa)	Elongation at Break *ε_t_* (%)	Izod Impact Strength *a_iN_* (kJ/m^2^)	Durometer Hardness *HDD*
PA1010	-	43 ± 1.2	1.4 ± 0.17	42 ± 5.9	5.2 ± 1.11	68 ± 1.0
HF	20	52 ± 2.6	2.4 ± 0.13	8 ± 1.6	3.3 ± 0.72	75 ± 0.5
HF-S1	20	53 ± 1.9	2.3 ± 0.07	8 ± 1.2	4.0 ± 0.60	77 ± 1.0
HF-S2	20	51 ± 3.0	2.4 ± 0.15	8 ± 2.4	3.6 ± 0.69	75 ± 1.0
HF-S3	10	53 ± 3.6	2.3 ± 0.20	11 ± 1.8	3.2 ± 0.78	76 ± 1.0
HF-S3	20	56 ± 1.5	2.7 ± 0.24	4 ± 1.0	2.8 ± 0.38	78 ± 0.5
HF-S3	30	64 ± 5.5	3.2 ± 0.31	4 ± 1.4	2.0 ± 0.47	77 ± 0.5
